# The predictive utility of functional status at discharge: a population-level cohort analysis

**DOI:** 10.1186/s12877-021-02652-6

**Published:** 2022-01-03

**Authors:** Mats L. Junek, Aaron Jones, George Heckman, Catherine Demers, Lauren E. Griffith, Andrew P. Costa

**Affiliations:** 1 Department of Medicine, McMaster University, 1280 Main Street West, Hamilton, ON L8S 4L8 Canada; 2 Department of Health Research Methods, Evidence, and Impact, McMaster University, Hamilton, Ontario Canada; 3 Schlegel Research Institute on Aging, Waterloo, Ontario Canada; 4 University of Waterloo, School of Public Health and Health Systems, Waterloo, Ontario Canada; 5 McMaster Institute for Research on Aging, Hamilton, Ontario Canada

**Keywords:** functional status, routinely-collected data, health services

## Abstract

**Background:**

Functional status is a patient-important, patient-centered measurement. The utility of functional status measures to inform post-discharge patient needs is unknown. We sought to examine the utility of routinely collected functional status measures gathered from older hospitalized patients to predict a panel of post-discharge outcomes.

**Methods:**

In this population-based retrospective cohort study, Adults 65+ discharged from an acute hospitalization between 4 November 2008 and 18 March 2016 in Ontario, Canada and received an assessment of functional status at discharge using the Health Outcomes for Better Information and Care tool were included. Multivariable regression analysis was used to determine the relationship between functional status and emergency department (ED) re-presentation, hospital readmission, long term care facility (LTCF) admission or wait listing (‘LTCF readiness’), and death at 180 days from discharge.

**Results:**

A total of 80 020 discharges were included. 38 928 (48.6%) re-presented to the ED, 24 222 (30.3%) were re-admitted, 5 037 (6.3%) were LTCF ready, and 9 047 (11.3%) died at 180 days. Beyond age, diminished functional status at discharge was the factor most associated with LTCF readiness (adjusted Odds Ratio [OR] 4.11 for those who are completely dependent for activities of daily living compared to those who are independent; 95% Confidence Interval [CI]: 3.70-4.57) and death (OR 3.99; 95% CI: 3.67-4.35). Functional status also had a graded relationship with each outcome and improved the discriminability of the models predicting death and LTCF readiness (p<0.01) but not ED re-presentation or hospital re-admission.

**Conclusion:**

Routinely collected functional status at discharge meaningfully improves the prediction of long term care home readiness and death. The routine assessment of functional status can inform post-discharge care and planning for older adults.

**Supplementary Information:**

The online version contains supplementary material available at 10.1186/s12877-021-02652-6.

## Background

Predicting health service use after discharge from acute hospital admissions is generally based on physiologic measurements such as age, diagnoses, or test results. Such parameters, however, are narrow definitions of health that do not fully reflect the patient as an independent human being. Functional status describes the ability of a patient to perform the daily activities required to meet their basic needs and maintain their health and well-being [1]. As a global measure, functional status is a patient-centred, patient-understood metric of health that integrates their overall physiologic capabilities and can be used to monitor effectiveness of therapy, quality of care, and inform discharge planning [2–5].

In studies of hospital inpatients, functional status is a robust predictor of post-discharge outcomes for older adults including admission to long-term care homes (LTCFs), mortality, and development of geriatric syndromes (e.g. falls and incontinence) [4, 6–9]. The value of functional status measures has also been established to have prognostic and therapeutic utility in individual diseases including heart failure, stroke, and cancer [10–12]. Despite this, functional data is often not collected for inpatients nor routinely considered by physicians, and consequently not included in administrative datasets used for population studies [13–15].

The Health Outcomes for Better Information in Care (HOBIC) initiative was a large-scale pilot program implemented in Ontario, Canada from 2008 to 2016 that sought to routinely capture data concerning patient function, self-care, symptom burden, and safety for all hospitalized patients at admission and discharge [16]. Using discharge functional status data collected during the program, we sought to examine its utility in predicting a panel of post-hospital discharge outcomes. We hypothesized that for all outcomes, discharge functional status will be an independent predictor positively associated with each outcome, and the strongest predictors of future placement on waitlist for or admission to an LTCF and death post-discharge.

## Methods

### Study Design

We conducted a retrospective cohort study of adults aged 65 or older who were discharged following an unplanned hospitalization in Ontario, Canada, from hospitals that participated in the HOBIC initiative between 2008 and 2016. Ontario is Canada’s most populous province, containing approximately 40% of the Canadian population and 268 publicly funded hospitals (27.8% of all hospitals in Canada) [17, 18].

This study was granted an exemption from ethics review as the use of the data in this project is authorized under section 45 of Ontario’s Personal Health Information Protection Act (PHIPA) and does not require review by a Research Ethics Board. We followed the Strengthening the Reporting of Observational Studies in Epidemiology and Reporting of Studies Conducted using Observational Routinely-Collected Health Data guidelines [19, 20].

### Data Sources

HOBIC was not evenly implemented across all hospitals in Ontario. As such, we defined three criteria to designate hospitals that completed an adequate number of HOBIC assessments to be included: 1) at least 100 HOBIC assessments were completed at the hospital; 2) at least five percent of all admissions and/or discharges had a HOBIC assessment completed; and 3) during each quarter that HOBIC assessments were being completed, during at least two of the three months of that quarter there were enough HOBIC assessments completed to be greater than or equal to 25% of overall mean monthly HOBIC assessments at that hospital.

Data were accessed at the Institute for Clinical Evaluative Sciences, an independent, non-profit research institute whose legal status under Ontario’s health information privacy law allows it to collect and analyze health care and demographic data, without consent, for health system evaluation and improvement [21]. Data was extracted for analysis on 12 August 2019.

### Study population

Participants were selected if they were discharged from the hospital during the dates that the HOBIC program was active (4 November 2008 to 18 March 2016) and had undergone an assessment of their functional status through the HOBIC tool. The following exclusion criteria were applied to address those who were expected to or had already experienced an outcome: age less than 65 on admission; planned admission; hospital transfer; admission from LTCF; patient undergoing dialysis or chemotherapy; patient discharged to a non-community location (e.g. LTCF, rehabilitation facility, or palliative care); patient discharged after being placed on an LTCF wait list. Multiple admissions from individuals were included provided that they had a new HOBIC assessment during the admission and were re-admitted from a community setting.

### Exposures

Within HOBIC, functional status was assessed by nurses trained on the use of the HOBIC instrument through the following basic activities of daily living (bADLs): bathing, hygiene, locomotion, toilet transfer, toileting, bed mobility, and eating. The bADLs were interRAI assessment system standards using an ordinal score set out by Morris, Fries & Norris [22, 23]. Where values were missing across two or fewer bADLs, missing values were imputed using the integer-rounded mean of present bADLs. ADL scores for patients were then compiled into a bADL hierarchy (ADLH) that stratified them into clinically meaningful phenotypes (Supplementary data Figure SF1) [23]. Pre-admission functional data, which has demonstrated prognostic utility in other cohorts, was unavailable, rendering functional trajectories unmeasurable. Discharge ADLH was treated as a categorical variable on a scale of zero to five (referent zero). HOBIC has demonstrable internal validity and interrater reliability [16].

Additional variables were collected across four areas: demographics (age, sex, if they lived in a rural location, and income quintile); continuity of primary care (usual provider index), number of visits to the primary care provider in the last year) [24–26]; index admission characteristics (length of stay, admission to the intensive care unit (ICU)); and burden of comorbidities (Charlson comorbidity index), diagnosis of asthma, chronic obstructive pulmonary disease, angina, coronary artery disease, heart failure, hypertension, type II diabetes mellitus type II, epilepsy, dementia, delirium, injurious falls, and/or stroke) [27, 28]. Variables were selected based on information from previous studies [4, 29, 30]. Detailed information concerning variables, exposure definitions and datasets can be found in Supplementary data tables ST1 and ST2; datasets were linked using unique encoded identifiers and analyzed at ICES. Age category (referent age 65-69) and income quintile (referent highest quintile) were treated as categorical variables, length of stay was treated as a continuous variable, all other variables were treated as binary variables.

### Outcomes

The following outcomes at 180 days after discharge from the index admission were used: Emergency department (ED) re-presentation, hospital re-admission, death, and a composite of being admitted to an LTCF or being placed on the wait list for an LTCF (‘LTCF readiness’) as in both circumstances the patient was sufficiently impaired that they required LTCF-level assistance. Follow-up was based on linkage to existing secondary records with complete capture. Information concerning outcome data sources can be found in supplementary data table ST1.

### Analysis

Descriptive analysis of outcomes was completed using mean and standard deviation as well as median and interquartile range where appropriate. Where a patient had multiple outcomes, each outcome was considered separately. Models predicting each outcome were constructed using multivariable logistic regression analysis. Given the high number of events for each outcome, all variables were able to be considered across each model.

The additive value of function in predicting each outcome was assessed by performing regressions including and excluding discharge ADLH and comparing Receiver-Operating Characteristics (ROC) using χ^2^. Goodness-of-fit was determined using the calibration plots of the models for each outcome stratified by age [31, 32]. Sensitivity analysis was performed using the bADL long form (the gross sum of a patient’s bADL scores), individual bADL scores, as well as the non-imputed data set where data was considered missing if any bADLs required to calculate the ADLH were missing.

All analyses were completed using SAS version 9.4 [33].

## Results

### Population Characteristics

A total of 53 (28.8%) public hospitals in Ontario completed sufficient HOBIC assessments to be included in the analysis, which allowed for the inclusion of 80,020 patient discharges across 73,813 patients (Supplementary figure SF2). The cohort was 48.7% male, with an average age of 77.9 ± 7.9. Within 180 days after discharge, 38,928 (48.6%) re-presented to the ED, 24,222 (30.3%) were re-admitted to hospital, 5,037 (6.3%) were LTCF ready and 9,047 (11.3%) died.

Patients who had the lowest rate of ICU admission, higher discharge ADLH and the longest length of stay were most likely to be LTCF ready; patients with the lowest discharge ADLH and shortest length of stay tended to have no outcome at 180 days (Table 1). Patients with the highest Charlson comorbidity index as well as prevalence of heart failure and or COPD died; patients with higher rates of dementia, delirium, stroke, and injurious falls were more frequently LTCF ready.

**Table 1:** Demographics, comorbidities, and index hospitalization characteristics stratified by individual discharge outcome

	All	ED re-presentation	Re-admitted to hospital	LTCF ready	Death	None
Patients (%)	80 020 (100.0)	38 928 (48.6)	24 222 (30.3)	5 037 (6.3)	9 047 (11.3)	35 831 (44.8)
Age**	75.7 (75.7-75.8)	76.4 (76.3-76.5)	76.5 (76.4-76.6)	81.5 (81.3-81.7)	77.8 (77.7-78.0)	74.8 (74.7-74.9)
Female	51.2	50.7	48.6	60.3	45.8	51.8
Income Quintile*	3 (2-4)	3 (2-4)	3 (2-4)	2 (1-4)	3 (2-4)	3 (2-4)
Lives Rurally	21.5	23.7	20.8	22.2	23.8	18.9
Family physician visits in last year**	0.88 (0.87-0.88)	0.90 (0.89-0.91)	0.82 (0.80-0.83)	0.62 (0.59-0.64)	0.60 (0.58-0.62)	0.91 (0.89-0.96)
Usual provider index**	0.38 (0.38-0.38)	0.35 (0.35-0.36)	0.35 (0.35-0.35)	0.40 (0.39-0.40)	0.34 (0.34-0.35)	0.40 (0.40-0.40)
days to outcome*	-	36 (11-87)	42 (14-92)	51 (23-99)	59 (27-106)	-
Comorbidities						
Charlson comorbidity index**	1.31 (1.30-1.33)	1.51 (1.49-1.52)	1.72 (1.69-1.74)	1.45 (1.41-1.49)	2.50 (2.45-2.54)	1.03 (1.02-1.04)
Angina	9.3	9.6	8.6	5.1	7.1	9.4
Asthma	0.8	0.7	0.7	0.5	0.4	0.9
Coronary Artery Disease	17.3	17.7	16.8	10.9	14.5	17.6
Heart Failure	13.0	15.6	17.7	15.7	21.6	10.1
COPD	10.9	12.5	13.2	10.7	15.5	9.4
Diabetes	22.8	24.4	25.9	23.1	25.3	21.2
Epilepsy	0.4	0.5	0.5	0.5	0.4	0.3
Hypertension	28.6	27.7	26.9	27.9	22.7	30.1
Stroke	2.4	2.1	1.9	3.8	1.7	2.6
Delirium	3.7	4.0	4.2	11.3	4.2	3.0
Dementia	1.1	1.1	1.2	5.3	1.4	0.8
Injurious Fall	6.1	5.0	4.4	11.8	3.6	7.0
Index hospitalization characteristics						
Length of stay*	5 (3-7)	5 (3-9)	5 (3-10)	8 (4-15)	6 (4-11)	4 (2-7)
Admitted to ICU	13.4	13.0	12.6	8.3	10.1	14.2
Discharge ADLH*	0 (0-2)	0 (0-2)	0 (0-2)	2 (0-4)	1 (0-3)	0 (0-1)

ICU = intensive care unit; ADLH = activities of daily living hierarchy; COPD = chronic obstructive pulmonary disease. *median and interquartile range;

**mean and 95% Confidence interval

### Functional Status and Outcomes

**Fig. 1:**
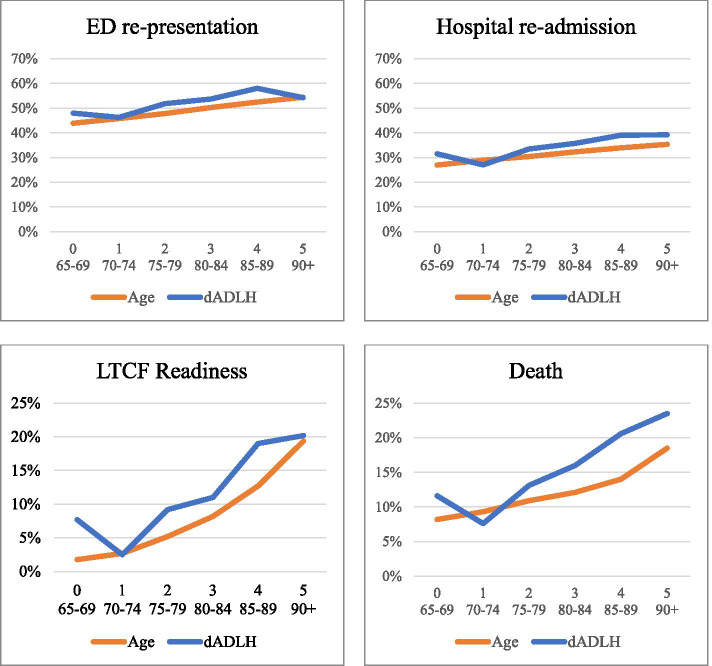
Proportion of patients in the cohort with each outcome by age and discharge ADLH (dADLH). 0 = independent; 1 = requires supervision; 2 = requires limited assistance; 3 = requires extensive assistance; 4 = requires maximal assistance; 5 = dependent. Patients who experienced more than one outcome were included in each

The most common functional status at discharge was functionally independent (38.6%). Increasing age and discharge ADLH were both associated with higher rates of each of the four outcomes (figure 1, supplementary table ST3). In those who were functionally dependent, 20.2% were LTCF ready and 23.5% died at 180 days, compared to 7.7% and 11.6%, respectively, of those who were functionally independent at discharge.

In unadjusted analysis, a discharge ADLH cut-off of rank 3 or higher (requiring extensive supports or more to perform bADLs) was associated with increased odds of each outcome (table 2). These results are similar to those found in the regression analysis; extensive or more supports were associated with increased risk of each outcome but were most potently associated with LTCF readiness (OR 4.96, 4.67 – 5.28) and Death (OR 3.04, 2.89 – 3.20).

**Table 2:** Predictive Utility of Discharge Functional Status (limited or no ADL support vs. extensive or more ADL) for each outcome using unadjusted analysis

Outcome	Sensitivity (95% CI)	Specificity (95% CI)	Odds Ratio (95% CI)	Positive LR (95% CI)	Negative LR (95% CI)
**ED** **re-presentation**	13.9% (13.5-14.2)	88.5% (87.6-89.4)	1.24 (1.19 – 1.30)	1.21 (1.17 – 1.25)	0.97 (0.97 – 0.98)
**Hospital** **re-admission**	16.0% (15.6-16.5)	88.8% (88.1-89.6)	1.52 (1.46 – 1.59)	1.44 (1.39 – 1.49)	0.95 (0.94 – 0.95)
**LTCF Readiness**	37.9% (36.5-39.2)	89.1% (88.4-89.7)	4.96 (4.67 – 5.28)	3.46 (3.32 – 3.61)	0.70 (0.68 – 0.71)
**Death**	26.9% (26.0-27.8)	89.2% (88.5-89.9)	3.04 (2.89 – 3.20)	2.49 (2.39 – 2.59)	0.82 (0.81 – 0.83)

LR = likelihood ratio

In adjusted analysis, age and functional status were most predictive of LTCF readiness or death at 180 days, though both were associated with increased odds of ED re-presentation and hospital re-admission as well (Table 3). Those who required extensive assistance or more had OR 4.11-4.75 for being LTCF ready as compared to those who were independent. Delirium, dementia, injurious falls, and seizures were all associated with higher odds of being LTCF ready; the Charlson comorbidity index was not.

**Table 3:** Adjusted Odds Ratios by outcome

	ED Re-presentation		Hospital Readmission		LTCF Readiness		Death	
	OR	95% CI	OR	95% CI	OR	95% CI	OR	95% CI
Age 70-74*	1.08	(1.03-1.13)	1.08	(1.02-1.13)	1.36	(1.15-1.61)	1.14	(1.05-1.25)
75-79*	1.19	(1.14-1.24)	1.18	(1.12-1.24)	2.52	(2.16-2.93)	1.38	(1.26-1.50)
80-84*	1.32	(1.26-1.38)	1.27	(1.21-1.34)	3.61	(3.12-4.17)	1.59	(1.47-1.73)
85-89*	1.48	(1.41-1.56)	1.39	(1.31-1.47)	5.06	(4.37-5.85)	1.81	(1.65-1.97)
≥90*	1.67	(1.57-1.78)	1.51	(1.41-1.62)	7.22	(6.21-8.39)	2.47	(2.24-2.73)
Sex (F v M)	0.97	(0.94-1.00)	0.86	(0.84-0.89)	1.16	(1.09-1.23)	0.76	(0.73-0.80)
Income Quintile 2 **	0.90	(0.86-0.94)	0.96	(0.91-1.00)	0.82	(0.75-0.89)	1.03	(0.96-1.11)
3 **	0.87	(0.83-0.9)	0.97	(0.93-1.02)	0.78	(0.71-0.85)	1.04	(0.97-1.12)
4 **	0.84	(0.8-0.87)	0.94	(0.89-0.98)	0.69	(0.62-0.76)	1.01	(0.94-1.09)
5 **	0.83	(0.79-0.86)	0.94	(0.89-0.98)	0.71	(0.65-0.78)	0.97	(0.9-1.04)
Lives Rurally	1.38	(1.33-1.42)	0.97	(0.93-1.01)	1.13	(1.05-1.22)	1.19	(1.12-1.26)
Usual Provider index	0.92	(0.88-0.96)	0.91	(0.87-0.96)	0.71	(0.63-0.79)	0.77	(0.71-0.84)
FP visits	1.08	(1.06-1.10)	0.95	(0.94-0.97)	0.86	(0.83-0.90)	0.78	(0.76-0.80)
ICU admit	0.92	(0.88-0.96)	0.91	(0.87-0.96)	0.71	(0.63-0.79)	0.77	(0.71-0.84)
Length of Stay	1.00	(1.00-1.00)	1.01	(1.00-1.01)	1.02	(1.01-1.02)	1.00	(1.00-1.00)
Discharge ADLH								
Independent	Referent		Referent		Referent		Referent	
Supervision	1.16	(1.10-1.22)	1.22	(1.16-1.30)	2.52	(2.27-2.80)	1.57	(1.44-1.71)
Limited Assist	1.24	(1.19-1.29)	1.33	(1.27-1.39)	2.86	(2.63-3.12)	1.87	(1.75-2.00)
Extensive Assist	1.42	(1.30-1.54)	1.44	(1.32-1.57)	4.75	(4.21-5.35)	2.46	(2.20-2.75)
Max Assist	1.27	(1.18-1.36)	1.52	(1.41-1.64)	4.90	(4.40-5.47)	2.95	(2.67-3.24)
Dependent	1.11	(1.04-1.19)	1.44	(1.34-1.54)	4.11	(3.70-4.57)	3.99	(3.67-4.35)
Comorbidities								
CCI	1.15	(1.14-1.17)	1.23	(1.22-1.25)	1.02	(0.99-1.04)	1.59	(1.57-1.61)
Angina	1.08	(1.01-1.16)	0.91	(0.84-0.98)	0.85	(0.71-1.02)	0.88	(0.77-0.99)
Coronary Artery Disease	0.95	(0.90-1.00)	0.91	(0.85-0.96)	0.82	(0.72-0.93)	0.80	(0.73-0.88)
Heart Failure	1.18	(1.12-1.23)	1.25	(1.20-1.31)	1.05	(0.96-1.15)	1.26	(1.18-1.34)
COPD	1.15	(1.10-1.20)	1.09	(1.04-1.14)	1.02	(0.93-1.13)	1.07	(1.00-1.14)
Delirium	1.07	(0.99-1.16)	0.96	(0.89-1.05)	1.88	(1.69-2.10)	0.74	(0.65-0.83)
Dementia	0.87	(0.76-1.00)	0.87	(0.75-1.00)	3.04	(2.59-3.57)	0.67	(0.54-0.82)
Diabetes	0.95	(0.91-0.99)	0.90	(0.87-0.94)	1.09	(1.00-1.18)	0.56	(0.53-0.60)
Injurious Fall	0.71	(0.67-0.76)	0.63	(0.59-0.68)	1.21	(1.10-1.34)	0.50	(0.44-0.56)
Hypertension	0.89	(0.86-0.92)	0.85	(0.82-0.88)	0.88	(0.82-0.95)	0.68	(0.64-0.72)
Seizure	0.73	(0.66-0.80)	0.62	(0.55-0.69)	1.41	(1.19-1.67)	0.43	(0.36-0.52)
Stroke	1.63	(1.30-2.05)	1.31	(1.03-1.66)	1.23	(0.80-1.88)	1.02	(0.70-1.48)

*compared to age 65-69; **compared to income quintile 1; FP = family physician; CCI = Charlton Comorbidity Index; COPD = Chronic Obstructive Pulmonary Disease

Patients who died had similar finding, however the association with age was less pronounced. Worsening functional status was progressively associated with increased odds of dying; those who were functionally dependent had an OR of 3.99 (3.67-4.35) as compared to those who were independent. Charlson comorbidity index demonstrated the greatest magnitude of association for those who died as compared to other outcomes, OR 2.50 (2.45-2.54).

### Predictive utility of models

Model ROCs demonstrated poor discriminability for ED re-presentation and hospital re-admission (ROC 0.621, 0.617-0.625 and 0.644, 0.640-0.648 respectively). LTCF readiness (0.819, 0.814-0.825) and death (0.782, 0.776-0.787) were predicted with reasonable discriminability. The addition of function to the ED or hospital re-admission models did not improve discriminability (p = 0.27 and 0.06 respectively) while significant improvements were seen in the discrimination of LTCF readiness and death (p < 0.01 for both). Calibration curves demonstrated goodness of fit across all outcomes (supplementary figure SF3).

### Sensitivity analysis

Sensitivity analysis demonstrated that when the sum of each bADL score or patient bADLs was used in place of the discharge ADLH there was no difference in results (p=0.26 for both sensitivity analyses). Analysis using a non-imputed dataset also yielded similar results.

## Discussion

We found that lower functional status at discharge was a leading predictor of LTCF readiness and death at 180 days post-discharge. Increasing age was the factor that most impacted outcomes beyond functional status. Our results suggest that routinely collected functional status at discharge meaningfully improves the prediction of post-discharge LTCF readiness and death, but not ED re-presentation or hospital re-admission. The routine assessment of functional status can inform ongoing health care needs, post-discharge service planning, and provide better targeting of care.

A ‘plateau’ effect was seen where those requiring maximal or greater assistance with bADLs were no more likely to be LTCF ready than those requiring extensive or maximal assistance, which was likely driven by patients dying or being discharged to a palliative setting (rather than a LTCF). Patients with independent functional status at discharge were more likely to experience an outcome than those who required supervision to complete ADLs. This may have represented preferential coding of patients as independent on discharge, that supervision provides a safety benefit, or both. The smaller magnitude of change in outcomes across functional status compared to age for ED and hospital re-admission reflects that function may be a poor discriminator of these outcomes as well as the general stochasticity of these events.

Across the cohort, several findings were seen that are reflective of previous analyses. Though there were high rates of ED re-presentation and hospital re-admission, similar rates have been seen in other studies [34, 35]. ICU admission was protective of each outcome; these patients had less comorbidity (lower Charlson comorbidity index, p<0.01), a greater chance of surviving severe illness (p<0.01), and were thus selected to be cared for there [36]. An increased usual provider index and number of recent family physician visits were protective for all except ED re-admission, reflecting that consistent primary care may prevent acute decompensation [24, 37]. Heart failure and COPD were associated with increased odds of each outcome except LTCF readiness, indicating the additional prognostic burden they carry.

More heterogeneity is seen when contextualizing the prognostic value of function within previous studies. In terms of re-hospitalization, there has been conflicting data; smaller studies have demonstrated that functional data is helpful. Larger, database driven models, however, have generally not included functional measures [5, 29, 38–40]. Smaller studies of ED re-presentation have also demonstrated the value of functional measurement in the prognostication of outcomes [41, 42]. A meta-analysis of factors contributing to LTCF readiness congruently found that requiring assistance with bADLs (1-2 bADLs OR 2.45, 2.02 – 2.97; 3 or more bADLs 3.25, 2.59 – 4.09) and prior nursing home use (OR 3.47, 1.88 – 6.37) were the factors most associated with LTCF admission [3]. There were several methods by which ADLs were assessed within the studies, suggesting that there can be flexibility in how function is measured. For mortality, function was only measured in non-database studies; where measured, functional deterioration was the greatest predictor of death [4].

Strengths and applicability of this study relate mainly to the cohort and data collection. The results were found within a large and diverse cohort using routinely collected data that minimizes selection bias and supports ‘real world’ applicability. The population are drawn from across a province with a population with significant sociodemographic and comorbid diversity. Our results are also novel as, to our knowledge, this is the only study to compare prognostic factors across different outcomes to date.

Collectively, these findings suggest that the greatest barrier to using functional measure within clinical care is the feasibility and routine practice of the collection of functional data itself. Future research should address this issue. While HOBIC data was collected by nurses during the program, its collection may be facilitated by looking to see if such information can be collected from assessments by physiotherapists and occupational therapists as well. Second, using such data to demonstrate how this data can change real world outcomes, such as ensuring senior friendly hospitals or reduced emergency applications to LTCFs, would reinforce the case for its routine collection [43].

Limitations to this work largely relate to the deployment of the HOBIC program. Despite the use of liberal hospital inclusion criteria in the study, most hospitals were deemed ineligible due to an insufficient number of HOBIC assessments completed. Included hospitals were mostly urban, limiting the generalizability of this data in more rural settings. There was also poor capture of some comorbidities including delirium and dementia (though reasonable estimates of coronary artery disease, hypertension, diabetes, and chronic obstructive pulmonary disease) [44–47]. Finally, this analysis does not include data concerning whether individuals received home care services post-discharge to support them remaining in the community. It was expected that the inclusion of such data would increase the magnitude of the association by demonstrating the necessity of these services for vulnerable individuals [48].

Functional status is an important predictor of LTCF readiness and death after acute hospitalization. Internal and external consistency of results validates the importance of assessing function in-hospital. Routinely collected functional status data has the potential to meaningfully inform future health care planning.

## Supplementary Information

**Additional file 1.**

## Data Availability

The data that support the findings of this study are available from ICES but restrictions apply to the availability of these data, which were used under license for the current study, and so are not publicly available. Data are however available from the authors upon reasonable request and with permission of the ICES. Protocol and statistical code are available on request from the main author. Parts of this material are based on data and/or information compiled and provided by CIHI. However, the analyses, conclusions, opinions and statements expressed in the material are those of the author(s), and not necessarily those of CIHI.

## Abbreviations

LTCF: long term care facility
HOBIC: Health Outcomes for Better Information in Care initiative
bADL: basic activities of daily living
ADLH: bADL hierarchy
ICU: intensive care unit
ED: emergency department
