# Supplementary material for: The predictive utility of functional status at discharge: a population-level cohort analysis
Source: BMC Geriatr. 2022 Jan 3;22:8. doi: 10.1186/s12877-021-02652-6 (PMC8722185; doi:10.1186/s12877-021-02652-6)
Supplement: Supplementary file 1 — Additional file 1. [file 12877_2021_2652_MOESM1_ESM.docx]

**Supplementary Data**

Figure SF1: Method of aggregating activities of daily living (ADLs) into a hierarchy. The four of the seven original ADLs used for the hierarchy are eating, hygiene, toileting, and locomotion. Adapted from (21).

| Variable | Database & Derivation |
| --- | --- |
| **Demographics** | |
| Age | RPDB in five year age brackets with all aged 90 or more consolidated |
| Sex | RPDB |
| Living in a rural location | RPDB |
| Local Health Integration Network (LHIN) | RPDB, the health administrative area that each individual resides in |
| Income quintile | RPDB, as estimated from postal code the individual resides in |
| **Functional Characteristics** | |
| Admission bADLs | HOBIC |
| Discharge bADLs | HOBIC |
| Continuity of Care | |
| Usual Provider Index (UPI) | OHIP, using known ICES Macro ^2^ |
| Visits to family physician in the last year | OHIP |
| **Admission Characteristics** | |
| Length of stay | DAD |
| Admission to ICU | DAD |
| Discharge Diagnosis | DAD |
| **Comorbidities & Ambulatory Care Sensitive Conditions** | |
| Charlson Comorbidity Index | DAD, using methods of Quan et al. ^3^ |
| History of: asthma, COPD, angina, CAD, heart failure, hypertension, diabetes, epilepsy, dementia, delirium, injurious falls, stroke | DAD; coded using methods outlined by Hux and Tang ^4^ and codes listed in supplemental data table ST2 |
| **Outcomes** | |
| ED re-presentation | NACRS |
| Hospital re-admission | DAD |
| LTCF readiness | CCRS |
| Death | RPDB |

Table ST1: sources and handling of individual variables used in cohort construction. RPDB = Registered Persons Database; OHIP = Ontario Health Insurance Program Database; DAD = Discharge Abstract Database; NACRS = National Ambulatory Care Reporting System; CCRS = Continuing Care Reporting System.

| Diagnosis | ICD-10 codes included |
| --- | --- |
| Diabetes | E11.0, E11.10, E11.11, E11.20, E11.21, E11.22, E11.23, E11.23, E11.33, E11.40, E11.41, E11.42, E11.50, E11.51, E11.52, E11.60, E11.61, E11.63, E11.64, E11.68, E11.70, E11.71, E11.78, E11.9 |
| Epilepsy / Seizure Disorders | G40.00, G40.10, G40.20, G40.3, G40.30, G40.31, G40.5, G40.50, G40.60, G40.7, G40.9, G40.90, G40.91, G41.0, G41.2, G41.9 |
| Delirium | F05.0, F05.1, F05.4, F05.8, F05.9 |
| Dementia | G30.1, G30.8, G30.9, G31.0, G31.2, G31.8, G31.9 |
| Congestive heart failure | I50.0, I50.1, I50.9, I51.0, I51.3, I51.4, I51.6, I51.7, I51.8, I51.9 |
| Hypertension | I10.0, I10.1, I11, I12, I13 |
| Angina | I20.0, I20.1, I20.80, I20.88, I20.9, I21.0, I21.1, I21.2, I21.3, I21.4, I 21.40, I21.41, I21.42, I21.49, I21.9 |
| Chronic Obstructive Pulmonary Disease | J44.0, J44.1, J44.8, J44.9 |
| Asthma | J45.00, J45.10, J45.90, J45.91 |
| Coronary Artery Disease | I20.0, I20.1, I20.80, I20.88, I20.9, I21.0, I21.2, I21.3, I21.4, I21.40, I21.41, I21.42, I21.49, I21.9, I25.0, I25.10, I25.11, I25.12, I25.13, I25.14, I25.15, I25.19, I25.2, I25.4, I25.5, I25.6, I25.8, I25.9 |

Table ST2: ICD-10 coding used for determining individual comorbidities.

| **Discharge ADLH** | **Number** | **Percent of cohort** |
| --- | --- | --- |
| Independent (0) | 53 120 | 65.9 |
| Supervision (1) | 6 213 | 7.7 |
| Limited Assistance (2) | 11 134 | 13.8 |
| Extensive Assistance (3) | 2 537 | 3.1 |
| Maximal Assistance (4) | 3 341 | 4.1 |
| Dependent (5) | 4 312 | 5.4 |

Table ST3: Frequencies of each discharge activity of daily living hierarchy (ADLH) within the cohort.

Figure SF2: Flowchart demonstrating the creation of the cohort and those included and excluded at each step of cohort creation.

|  |  |
| --- | --- |
|  |  |

Figure SF3: Calibration curves across age brackets demonstrating observed versus predicted events for each outcome using the multivariate regression.

1. Morris JN, Fries BE, Morris SA. Scaling ADLs within the MDS. *The Journals of Gerontology: Series A*. 1999;54(11):M546-M553.

2. Jaakkimainen L, Upshur R, Klein-Geltink J, et al. Primary care in ontario: ICES atlas. *Toronto: Institute for Clinical Evaluative Sciences*. 2006.

3. Quan H, Sundararajan V, Halfon P, et al. Coding algorithms for defining comorbidities in ICD-9-CM and ICD-10 administrative data. *Med Care*. 2005:1130-1139.

4. Hux JE, Tang M. Patterns of prevalence and incidence of diabetes. In: Hux JE, Booth GL, Slaughter PM, Laupacis A, eds. *Diabetes in ontario. an ICES practice atlas.* Toronto, ON: Institute for Clinical Evaluative Sciences; 2003.
